# Small molecules derived carbon dots: synthesis and applications in sensing, catalysis, imaging, and biomedicine

**DOI:** 10.1186/s12951-019-0525-8

**Published:** 2019-08-26

**Authors:** Anirudh Sharma, Joydeep Das

**Affiliations:** School of Chemistry, Shoolini University of Biotechnology and Management Sciences, Bajhol, PO Sultanpur, Solan, HP 173229 India

**Keywords:** Carbon dots, Biomedical applications, Hydrothermal/microwave synthesis, Photocatalysis, Sensing

## Abstract

Carbon dots (CDs) are the new fellow of carbon family having a size less than 10 nm and attracted much attention of researchers since the last decade because of their unique characteristics, such as inexpensive and facile synthesis methods, easy surface modification, excellent photoluminescence, outstanding water solubility, and low toxicity. Due to these unique characteristics, CDs have been extensively applied in different kind of scientific disciplines. For example in the photocatalytic reactions, drug-gene delivery system, in vitro and in vivo bioimaging, chemical and biological sensing as well as photodynamic and photothermal therapies. Mainly two types of methods are available in the literature to synthesize CDs: the top-down approach, which refers to breaking down a more massive carbon structure into nanoscale particles; the bottom-up approach, which refers to the synthesis of CDs from smaller carbon units (small organic molecules). Many review articles are available in the literature regarding the synthesis and applications of CDs. However, there is no such review article describing the synthesis and complete application of CDs derived from small organic molecules together. In this review, we have summarized the progress of research on CDs regarding its synthesis from small organic molecules (bottom-up approach) via hydrothermal/solvothermal treatment, microwave irradiation, ultrasonic treatment, and thermal decomposition techniques as well as applications in the field of bioimaging, drug/gene delivery system, fluorescence-based sensing, photocatalytic reactions, photo-dynamic therapy (PDT) and photo-thermal (PTT) therapy based on the available literature. Finally, the challenges and future direction of CDs are discussed.

## Introduction

Nano-crystals of carbon materials having dimensions smaller than 10 nm is known as carbon quantum dots (CDs) [1]. They exhibit different size reliant optical properties such as photoluminescence, chemiluminescence, electrochemical luminescence and photoinduced electron transfer [1, 2]. Besides, the high aqueous dispersibility, biocompatibility, good elasticity in modification, high resistance to photobleaching and chemical inertness make it well applicable in bio-imaging [3, 4], bio-sensing [5, 6], chemical-sensing [7], and biomedical applications [8]. Being a new kind of fluorescent nanomaterial and having excellent biocompatibility, CDs are widely used in the area of bio-imaging both in vitro and in vivo and in diagnosis purposes [9], Photothermal as well as photodynamic therapy and drug/gene delivery carriers [10, 11]. CDs could also been applied for the determination of cellular levels of biomolecules and ions (bio-sensor), such as Cu^2+^ [12], Hg^2+^ [13], NO_3_^−^ [14], C_6_H_12_O_6_ [15], pH [16], H_2_O_2_ [17], etc. CDs could also act as a promising photocatalyst after co-doping with heteroatoms, such as nitrogen, phosphorus, sulfur, and certain metal ions, such as Cu, Zn, Ti, etc. Incorporation of these elements improves the electron-donation/acceptance ability of the CDs and promotes redox reaction on the surface of CDs [18]. These properties of CDs are being employed for wastewater treatment and hydrogen generation [19, 20].

In 2004, during electrophoretic purification of single-walled carbon nanotubes (SWCNTs) fluorescent carbon nanoparticles were accidentally discovered by Xu et al. [21], and 2 years later Sun et al. synthesized CDs from graphite powder and cement using a laser ablation technique [22]. Mainly two types of methods are available in the literature for the synthesis of CDs; top-down approach and bottom-up approach. Top-down approach refers to breaking down larger carbon structures, such as carbon nanotubes and graphite into smaller carbon structures having dimensions less than 10 nm using arc discharge, laser ablation and electrochemical methods [23]. One such electrochemical method was adopted by Wang et al. for the synthesis of CDs from MWCNTs [24]. Bottom-up approach refers to the synthesis of CDs from smaller carbon units (small organic molecules) by electrochemical/chemical oxidation, laser ablation, hydrothermal/solvothermal treatment, microwave irradiation, ultrasonic treatment, and thermal decomposition techniques [25]. For example, Zhu et al. synthesized CDs via by heating a solution of poly (ethylene glycol) and saccharide in 500 W microwave oven for 2 to 10 min [26].

Various chemical precursors have been identified as the source of CDs, such as citric acid [27], glycerol [28], l-ascorbic acid [29], glucose [30], citric acid-urea [31] and thiourea [32]. To convert these precursors into fluorescent CDs various synthetic processes are used, such as ultrasonication [33], simple heating [34], arc discharge [35], solvothermal [36], hydrothermal [37–39], chemical oxidation [40], and laser ablation [41]. Plentiful efforts have been made to expand the usability of CDs to fulfill the growing demand for high-performance techniques, such as bio-imaging, drug-gene delivery, chemical sensing, as well as photocatalysis. However, it is also necessary to regulate the dimensions of CDs during its synthesis to attain uniform properties for a particular application. A large number of reports established the methods of purifying the as-prepared CDs via post-treatment, for example, centrifugation, filtration, gel-electrophoresis and column chromatography [42, 43]. Besides, monitoring the dimensions of CDs during its formation is also preferred.

In the past few years, many review articles have been published on the synthesis, properties and applications CDs which are derived from either small organic molecules (chemical) or green sources [44]. However, the number of review articles on CDs, which describe its synthesis only from chemical precursors and applications, like fluorescence-based sensing, bio-imaging, photocatalysis, drug/gene delivery carriers, photothermal as well as photodynamic therapy is still limited, and this article made an effort to fill the gap. In this current review article, we have described the recent progress of small molecule-derived CDs in the field of biomedical as well as chemical applications to date and their future perspective.

## Methods of synthesis of CDs

**Fig. 1:**
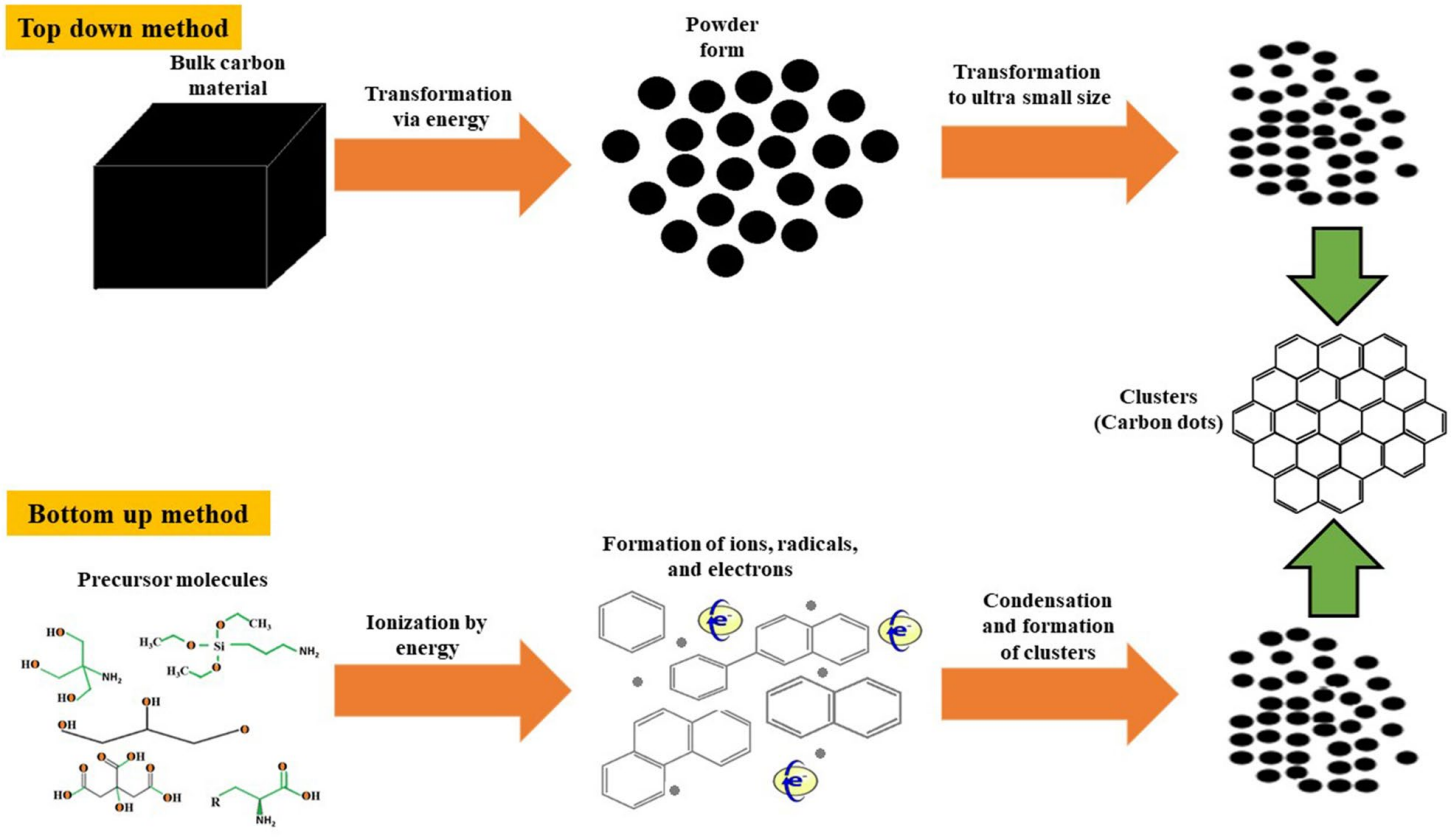
General synthesis methods of CDs. Bottom up approach: CDs are synthesized from smaller carbon units (small organic molecules) via applying energy (electrochemical/chemical, thermal, laser, etc.). The source molecules are getting ionized, dissociated, evaporated or sublimated and then condensed to form CDs. Top down approach: CDs are synthesized by transformation of larger carbon structures into ultra-small fragments via applying energy (thermal, mechanical, chemical, ultrasonic, etc.)

CDs can be synthesized mainly via two routes: (i) top-down approach and (ii) bottom-up approach (Fig. 1). Top-down approach refers to breaking down larger carbon structures via chemical oxidation, discharge, electrochemical oxidation, and ultrasonic methods [25]. However, drawbacks of this approach includes the requirement of expensive materials, harsh reaction conditions, and long reaction time [45]. On the other hand, the bottom-up approach refers to the conversion of smaller carbon structure into CDs of the desired size. This bottom-up approach is consisting of hydrothermal treatment, ultrasonic treatment, thermal decomposition, pyrolysis, carbonization, microwave synthesis and solvothermal method to synthesize CDs. Here we will discuss both the top-down and bottom-up approach for the synthesis of CDs obtained from small organic molecules.

### Top-down approach

Table 1 summarizes the several methods which are used for CD synthesis via the Top-down approach.

**Table 1:** Synthesis of CDs from small organic molecules via a top-down approach

S. No.	Source	Method of preparation	Doping (d)/surface passivating (p) agent	Color	Size (nm)	Refs. No.
1.	Carbon nanotube	Electrochemical synthesis	–	Blue	2.8 ± 0.5	[26]
2.	Carbon soot	Chemical oxidation	–	Green	2–6	[40]
3.	Carbohydrates	Chemical oxidation	(TTDDA) 4,7,10-trioxa-1,13-tridecanediamine (P)	Red, blue, green and yellow	5	[46]
4.	Low-molecular-weight alcohols	Electrochemical synthesis	–	Red and blue	2.1, 2.9, 3.5, and 4.3	[47]
5.	Sodium citrate and urea.	Electrochemical synthesis	–	Blue	1.0–3.5	[48]
6.	Graphite electrode	Electrochemical synthesis	–	Bright yellow	4 ± 0.2	[49]
7.	Toluene	Laser ablation	–	Red, black and blue	2–3.9, 3–10.0, 10–17.2 and 13–20.5	[41]
8.	Graphite powders	Laser ablation	–	Red, black and blue	1.5, 1.6, and 1.8	[50]
9.	Ascorbic acid and ammonia	Ultrasonic treatment	N (d)	Blue, green	3.36	[51]
10.	Oligomer polyamide resin	Ultrasonic treatment	Silane coupling agent (p)	Bright white	2–4	[33]

#### Electrochemical/chemical oxidation

Electrochemical/chemical oxidation is the most common top-down synthetic route for the synthesis of CDs, because of several remarkable advantages, such as high yield, high purity, low cost, and easy control over size. However, only a few articles are reported, so far regarding the electrochemical oxidation of small molecules to synthesize CDs. Zhou et al. demonstrated the first synthesis of CDs from carbon nanotube via electrochemical oxidation method [26]. Later, Ray et al. used carbon soots as the carbon source for the synthesis of CDs, and this approach can be used for the mg scale synthesis of CDs [40].

Peng et al. fabricated TTDDA passivated CDs with an average size of 5 nm from carbohydrates by dehydrating with conc. H_2_SO_4_ [46]. Fabrication of CDs from low molecular-weight alcohols was reported by Deng et al. [47]. They observed that the diameter of CDs was highly dependent upon applied potentials [47]. Hou et al. synthesized CDs from sodium citrate, and urea and the observed diameter of the CDs were in the range of 1.0 to 3.5 nm with 11.4% quantum yield (QY) [48]. Liu et al. used graphite electrode as the carbon source to synthesize CDs with an average diameter of 4 ± 0.2 nm [49].

#### Laser ablation

Laser ablation is another standard method used by the researchers for the synthesis of CDs. Yu et al. prepared CDs using toluene as the carbon source via laser irradiation technique. They controlled the size of CDs using laser furnace [41]. Nguyen et al. reported the synthesis of CDs from graphite powders via femtosecond laser ablation. They observed that the size of CDs and photoluminescence properties could easily be controlled by changing the parameters including spot size, irradiation time and laser fluence. Smaller size CDs can be synthesized by increasing the irradiation time [50].

#### Ultrasonic treatment

Ultrasonic treatment is also a very convenient method as the large carbon materials can be broken down by the action of very high energy of ultrasonic sound wave. Wang et al. synthesized N-doped CDs from ascorbic acid and ammonia via ultrasonic treatment [51]. Dang et al. fabricated CDs using oligomer-polyamide resin as the carbon source by ultrasonic treatment. The as-prepared CDs were well dispersed, had low crystallinity, and functional groups at the surface [33].

### Bottom-up approach

#### Hydrothermal synthesis

Hydrothermal synthesis method is being used by most of the researchers as a cheap, eco-friendly and low-cost route to synthesize CDs from saccharides, amines, organic acids and their derivatives (Table 2) [13, 52]. Zhang et al. synthesized fluorescent CDs for the first time from small organic compound, l-ascorbic acid via hydrothermal method. They heated the aqueous solution ascorbic acid at a constant 180 °C temperature for 4 h in an autoclave and purified the water-phase solution via dialysis using 8000–14,000 MWCO membrane. The as-prepared CDs had a diameter of 2 nm [53]. Qu et al. synthesized CDs from dopamine using this method. They heated the aqueous solution of dopamine at 180 °C temperature for 6 h in an autoclave and purified via centrifugation. The synthesized CDs were mostly spherical with an average diameter of about 3.8 nm [37]. Li et al. fabricated water soluble Nitrogen-doped CDs using ammonium citrate and ethylenediamine. They heated the aqueous solution of ammonium citrate and ethylenediamine at a constant 200 °C temperature for 5 h. The average diameter of the N-CDs was found to be 4.8 nm with a quantum yield of (QY) 66.8% [54]. Some researchers have also used amino acids as CDs source. Zeng et al. synthesized N, S co-doped CDs (N, S-CDs) having size ~ 2.6 nm from l-serine and l-cystine under hydrothermal reaction conditions [55]. Li et al. used citric acid and poly(ethylenimine) (PEI) as raw materials and synthesized CDs with an average diameter of ~ 4.5 nm and 48.3 ± 5.3% of QY by heating the aqueous solution of citric acid and PEI at 110 °C for 2 h [56]. The use of this method to synthesize CDs from small molecules has also been reported by several other researchers [6, 13, 38, 54, 55, 57–70].

**Table 2:** Synthesis of CDs from small organic molecules via hydrothermal treatment

S. No.	Source	Method of preparation	Doping (d)/surface passivating (p) agent	Color	Size (nm)	Refs. No.
1.	l-Ascorbic acid	Hydrothermal treatment	–	Violet	2	[53]
2.	Glucosamine HCl	Hydrothermal treatment	Glucosamine HCl (d)	Green	15–70	[57]
3.	Glucose, monopotassium phosphate	Hydrothermal treatment	–	Violet	1.83–3.83	[57]
4.	Dopamine	Hydrothermal treatment	–	Blue, yellow, green	3.8	[37]
5.	Sodium citrate	Hydrothermal treatment	–	Blue	1.59	[13]
6.	Citric acid and ethylene diamine	Hydrothermal treatment	–	Blue	2–6	[58]
7.	bPEI, ammonium persulfate	Hydrothermal synthesis	bPEI	Blue	3–4	[59]
8.	Streptomycin	Hydrothermal treatment	–	Violet	2.97	[60]
9.	Histidine, NaOH	Hydrothermal treatment	–	Blue	3–5	[61]
10.	Ammonium citrate, ethylenediamine	Hydrothermal treatment	N (d)	Blue	4.8	[54]
11.	l-Serine, l-cystine	Hydrothermal treatment	N, S (d)	Orange	2.6	[55]
12.	1-Octadecane 1-hexadecylamine, citric acid	Hydrothermal synthesis	Dihydrolipoic acid (p)	Yellow	6–8	[62]
13.	Citric acid	Hydrothermal treatment	Isoleucine (d)	Violet	6–15	[63]
14.	Ammonium citrate	Hydrothermal treatment	Ethylene diamine (d)	Indigo	4.8	[54]
15.	Citric acid, ethanediamine	Hydrothermal method	–	Violet	< 5	[64]
16.	l-Serine, l-cystine	Hydrothermal treatment	N, S (d)	Orange	2.6	[55]
17.	Citric acid, GSH	Hydrothermal treatment	–	Blue	2.5–3	[65]
18.	1-Octadecane 1-hexadecylamine, citric acid	Hydrothermal synthesis	Dihydrolipoic acid (p)	Yellow	6–8	[62]
19.	Citric acid, NaOH	Hydrothermal treatment	–	Green	11.3	[66]
20.	Citric acid, NH_3_·H_2_O	Hydrothermal treatment	N (d)	Blue	2	[6]
21.	Folic acid, phosphoric acid	Hydrothermal treatment	Folic acid, phosphoric acid (d)	Indigo	13.2 ± 1.6	[67]
22.	Glucose	Hydrothermal treatment	–	Blue	1.65	[68]
23.	Sodium nitrate, histidine	Hydrothermal treatment	–	Indigo	1.5	[69]
24.	l-Phenylalaninol	Hydrothermal carbonization	–	Violet	2.8	[38]
25.	Folic acid, phosphoric acid	Hydrothermal treatment	Folic acid, phosphoric acid (d)	Indigo	13.2 ± 1.6	[67]
26.	APTS (3-Aminopropyl)triethoxysilane), Glycerol	Hydrothermal synthesis	–	Violet	9 ± 0.5	[70]
27.	Citric acid, PEI (polyethyleneimine)	Hydrothermal treatment	–	Blue	4.5	[56]

#### Microwave-assisted synthesis

Microwave assisted synthesis is a fast and low-cost method to synthesize CDs via the irradiation of electromagnetic radiations having a wavelength ranging from 1 mm to 1 m through the reaction mixture containing the precursor molecules (Table 3) [71, 72]. Zhu et al. synthesized fluorescent CDs having size ~ 3.7 nm using microwave irradiation for the first time. They heated the aqueous solution of saccharides and polyethylene glycol in a domestic microwave oven (500 W) for nearly 3 min [73]. Liu et al. synthesized multicolor photoluminescence CDs with an average size of ~ 5 nm using glycerol as the carbon source and 4,7,10-trioxa-1,13-tridecanediamine (TTDDA) as the passivating agent [9]. Wang et al. synthesized water-soluble CDs by one-step microwave assisted pyrolysis of citric acid. They used tryptophan (Trp) as both a passivating agent and nitrogen source. They heated the aqueous solution of citrate and L-Trp in a microwave oven (700 W) for 3 min, and removed the large particles by centrifugation at 10,000 rpm to get CDs with the size of ~ 2.6 nm [74]. Kiran et al. used citric acid as a carbon source and 3-aminophenyl boronic acid as the passivation agent to fabricate CDs. They heated the aqueous solution of citric acid, and 3-aminophenyl boronic acid in a microwave oven (1200 W) for 4 min and the average diameter of the obtained CDs was ranging from 2 to 5 nm [75]. Recently Cao et al. (2018) synthesized CDs from the aqueous solution of glucose and arginine using microwave-assisted pyrolysis in a microwave oven (700 W) for near about 10 min. The average diameter of the as obtained CDs was between 1 and 7 nm [76]. Several other researchers have also reported the microwave-assisted the synthesis of CDs [27, 31, 77–81].

**Table 3:** Synthesis of CDs from small organic molecules via microwave treatment

S. No.	Source	Method of preparation	Doping (d)/surface passivating (p) agent	Color	Size (nm)	Refs. No.
1.	Saccharides and polyethylene glycol	Microwave synthesis	–	Blue	3.7	[73]
2.	Citric acid	Microwave synthesis	Tryptophan (d)	Indigo	2.6	[74]
3.	Glycerol	Microwave synthesis	TTDA (p)	Blue, turquoise, green, jacinth and red	5	[9]
4.	Carbohydrates and inorganic salts	Microwave synthesis	–	Blue, green, yellow, red	2.1	[77]
5.	Citric acid	Microwave synthesis	Tryptophan (d)	Indigo	2.6	[78]
6.	Citric acid	Microwave synthesis	RNase A (d)	Blue	25–45	[79]
7.	Citric acid, urea	Microwave synthesis	Boric acid, (d)	Indigo	2–6	[31]
8.	Citric acid	Microwave synthesis	3-Aminophenyl boronic acid (p)	Indigo	2–5	[75]
9.	Citric acid, urea	Microwave-assisted synthesis	–	Green	2–6	[27]
10.	Triammonium citrate	Microwave irradiation	–	Indigo	6.5	[80]
11.	Glycerol	Microwave pyrolysis	PEI (d, p)	Blue	9 ± 1.1	[81]
12.	Arginine and glucose	Microwave synthesis	–	Blue	1–7	[76]

#### Thermal decomposition

Researchers have also used the thermal decomposition technique as another standard bottom-up method to synthesize CDs (Table 4). In ordinary thermal decomposition, a substance or compound decomposes chemically by the action of heat. Thermal decomposition reactions are generally endothermic. This type of decomposition reactions are either irreversible (decomposition of starch, proteins) or reversible (decomposition of ammonium chloride, limestone). This method offers various advantages, such as easy to operate, less time consuming, low cost, and large scale production [45]. Wang et al. synthesized highly luminescent CDs by the thermal decomposition of citric acid as the carbon source and organosilane, *N*-(β-aminoethyl)-γ-aminopropyl methyl dimethoxy silane (AEAPMS) as the passivation agent. They heated the reaction mixture at 240 °C for only 1 min, and the observed diameter of CDs was ~ 0.9 nm [82]. Later, Wang et al. synthesized CDs by this method from citric acid. They heated citric acid on a hot plate at 200 °C for 30 min; neutralized with sodium hydroxide solution, and finally dialyzed for purification. The size of CDs was observed within the range from 0.7 to 1 nm [34]. These CDs showed both excitation-dependent as well as independent photoluminescent (PL) properties, and different QY depending on different synthesis conditions. Wan et al. used the thermal decomposition of 1-butyl 3-methyl imidazolium bromide and l-cysteine for the synthesis of CDs at 240 °C. AFM study showed that the height of the CDs was ranging from 1.0 to 3.5 nm [83]. Some other researchers also reported the synthesis of CDs from small organic molecules via this method [84–86].

**Table 4:** Synthesis of CDs from small organic molecules via thermal decomposition, carbonization, pyrolysis, solvothermal, and ultrasonic treatment

S. No.	Source	Method of preparation	Doping (d)/surface passivating (p) agent	Color	Size (nm)	Refs. No.
1.	Citric acid, *N*-(β-Aminoethyl)-γ-aminopropyl methyl dimethoxy silane	Thermal decomposition	AEAPMS (P)	Blue	0.9	[82]
2.	Citric acid	Thermal decomposition	DETA (p)	Blue	3–5.5	[84]
3.	Citric acid	Thermal decomposition	Ruthenium (III)	Blue	6.8 ± 2.3	[85]
4.	Citric acid	Thermal decomposition	–	Blue	0.7–1.0	[34]
5.	l-Cysteine	Thermal decomposition	1-butyl 3-methyl imidazolium bromide	Blue, yellow, red, green	1.0–3.5	[83]
6.	Citric acid	Thermal treatment	Dicyanamide (d)	Green	8–16	[86]
7.	Glucose	Carbonization	Ethylene diamine (d), phosphoric acid (p)	Green	1–7	[87]
8.	Citric acid	Carbonization	–	Blue	4.8–9	[88]
9.	6-*O*-(*O*–*O*-dilauroyl-tartaryl)-d-glucose	Carbonization	–	Green	2.4 ± 0.5	[89]
10.	Tris base, betaine Hcl	Pyrolysis	Gadopetetic acid (d)	Purple, Green	3.2	[31]
11.	GDs	Pyrolysis	l-glutamic acid	Blue, green and red	4.66–1.24	[17]
12.	d-Glucose	Pyrolysis	l-Aspartic acid (d)	Yellow	2.28 ± 0.42	[90]
13.	Sodium alginate	Pyrolysis	–	Blue	< 10	[91]
14.	Citric acid	Pyrolysis	Diethylenetriamine (p)	Indigo	5–8	[11]
15.	CCl_4_, NaNH_2_	Solvothermal method	N (d)	Blue, cyan, kelly, and yellow	3.3	[36]
16.	SiCl_4_, hydroquinone	Solvothermal treatment	Si (d)	Blue	7 ± 2	[29]
17.	hydroquinone	Solvothermal method	BBr_3_ (d)	Blue	16	[15]
18.	Glucose, HCl/NaOH	Ultrasonic treatment	–	Blue	< 5	[30]
19.	Active carbon, H_2_O_2_	Ultrasonic treatment	–	Blue, green, yellow, red	5–10	[92]

#### Carbonization synthesis

Carbonization of the precursor molecules is one of the best, inexpensive, simple, and ultrafast one-step methods to fabricate CDs (Table 4). Carbonization is a chemical process in which solid residues with higher content of carbon are formed from organic materials by prolonged pyrolysis in an inert atmosphere. Wei et al. synthesized N-doped CDs using this ultrafast carbonization method within 2 min from glucose as a carbon source, and ethylenediamine as the nitrogen source. The observed size of the CDs was in the range of 1 to 7 nm with 48% of QY [87]. Wang et al. fabricated blue luminescent thermally-reduced CDs (t-CDs) with size ranging from 4.8 to 9 nm using citric acid carbonization. They used thermogravimetric analyzer for thermal reduction of CDs, which resulted in the five times increment of QY compared with non-reduced-CDs [88]. Dolai et al. synthesized CDs–aerogel matrix using 6-*O*-(*O*-*O*-dilauroyl-tartaryl)-d-glucose as the carbon source. The observed diameter of nanoparticles was ~ 2.4 ± 0.5 nm [89].

#### Pyrolysis synthesis method

Pyrolysis method for the synthesis of CDs from precursor molecules is also preferred by some researchers (Table 4). Pyrolysis is an irreversible thermal decomposition reaction in which decomposition of organic materials take place in an inert atmosphere. It involves physical as well as chemical changes in organic materials resulting in solid residue containing carbon. Generally pyrolysis takes place at very high temperature and under controlled pressure. Bourlinos et al. synthesized Gd(III)-doped CDs having diameter ~ 3.2 nm with dual fluorescence via pyrolysis method. They prepared a mixture of tris(hydroxymethyl) aminomethane (Tris base), gadopentetic acid, and betaine hydrochloride to fabricate Gd(III)-CDs followed by the pyrolysis at 250 °C temperature [31]. Zheng et al. synthesized a new type of CDs using l-aspartic acid and d-glucose as source molecules via a straight forward pyrolysis method. They prepared the solution of l-aspartic acid and d-glucose in aqueous NaOH and heated it at 200 °C for 20 min. The observed average diameter of CDs was 2.28 ± 0.42 nm [90]. Feng et al. synthesized CDs from citric acid via thermal pyrolysis method. They used diethylenetriamine as the passivation agent, and according to TEM results, the size of CDs was ranging from 5 to 8 nm [11].

#### Solvothermal method

There are some reports where researchers used solvothermal method for the synthesis of CDs from small organic molecules as the carbon source (Table 4). For example, Zhang et al. (2012) synthesized N-doped CDs through the solvothermal route using CCl_4_ as carbon and NaNH_2_ as a nitrogen source. The as-prepared CDs were crystalline and had graphite-like structure with an average size of 3.3 nm and height ranging from 0.5 to 5 nm [36]. Qian et al. synthesized Si-doped CDs via this method using SiCl_4_ and hydroquinone. They heated the mixture of SiCl_4_ and hydroquinone in acetone within a stainless steel autoclave at 200 °C for 2 h. The observed diameter of the Si-doped CDs was 7 ± 2 nm [29]. Shan et al. used one-pot solvothermal method for the synthesis of Boron-doped CDs. They used hydroquinone as the precursor of carbon and BBr_3_ as the source of boron. The average size of the as-prepared B-doped CDs was ~ 16 nm [15].

#### Ultrasonic treatment

There are limited numbers of studies, where researchers used ultrasonic treatment method to synthesize CDs (Table 4). For example, Li et al. [30] synthesized water-soluble fluorescent CDs having a size in the range of 5–10 nm through the acid assisted ultrasonic treatment of glucose. In the same year, Li et al. synthesized water-soluble fluorescent CDs from activated carbon using one-step H_2_O_2_ assisted ultrasonic treatment method. According to the TEM results, the average size of CDs was ranging from 5 to 10 nm, and the surface of CDs was rich in hydroxyl groups [30, 92].

## Applications of CDs

### Bioimaging

**Fig. 2:**
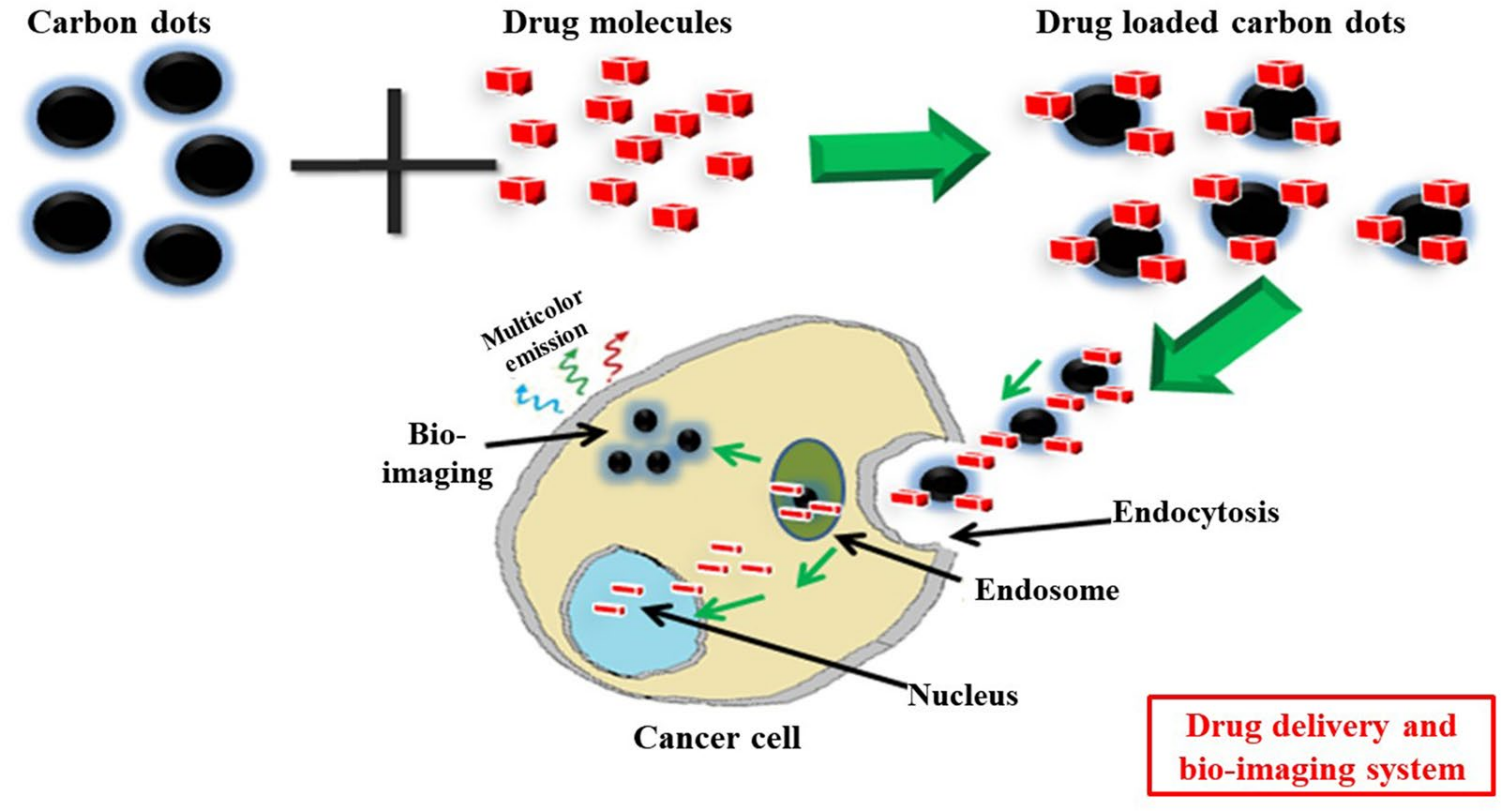
General mechanism of image guided drug delivery via CDs: drug loaded CDs enter into the cells, and deliver the drug to nucleus. Also, the intrinsic multicolor fluorescence nature of CDs helps in tracking the drug delivery pathway, and cellular imaging

CDs are considered as a potential candidate for bioimaging application due to its unique fluorescent nature, high photobleaching resistivity, less cytotoxicity, and better aqueous solubility (Fig. 2 and Table 5) [40, 93]. In the beginning, Ray et al. prepared the water-soluble, blue and yellow fluorescent CDs from carbon soot and nitric acid. The as-prepared CDs entered into the HepG2 cells and used for bio-imaging [40]. Qiao et al. used TTDDA (4,7,10-trioxa-1,13-tridecanediamine) passivated CDs for imaging of COS-7 cells [94]. After that Zhu et al. (2011) synthesized highly fluorescent graphene dots (GDs) via a one-step solvothermal route from graphene oxide for excitation-dependent fluorescent bioimaging of MG-63 cells [93]. Wang et al. fabricated biocompatible CDs/silica (silica-encapsulated CDs) nanoparticles having a size of 12 nm via decomposition pyrolysis method and applied them for bioimaging of BGC823 cells [82]. Yang et al. used glucose derived green florescent CDs as the bioimaging agent for labeling HepG2 cells. They also studied the intracellular localization of the CDs via counterstaining with 40,6-diamidino-2-phenylindole (DAPI). They showed cytoplasmic localization of the CDs surrounding the nucleus [52]. Peng et al. prepared water-soluble florescent CDs of carbon fibers for bioimaging of breast T47D cancer cells [95]. Carbon nanotubes and graphite derived yellow fluorescent CDs was used by Tao et al. for in vivo bioimaging and labeling purposes. A nude mouse was injected with CDs-M (CDs made from MWNTs) at three regions on its back-side intravenously, and the images were taken by the Maestro optical imaging system. They observed that the CDs were mainly accumulated within the liver and spleen. However, after short term exposure CDs were mainly accumulated within the kidney, indicating the urinary clearance of the CDs [3]. Zhang et al. used electrochemical method for synthesizing yellow fluorescent CDs with a diameter of 5 to 10 nm from graphite rods followed by reduction with hydrazine and applied for bioimaging of human lung A549 and breast MCF-7 carcinoma cells [96]. Dong et al. used CDs (15 nm) obtained via chemical oxidation of XC-72 carbon black for labeling MCF-7 breast cancer cells [97]. In the same year (2012) some other researchers also synthesized fluorescent CDs for bioimaging and labeling purposes [9, 57, 98–100]. After that in 2013 Pan et al. fabricated CDs for imaging HeLa cell. The CDs (3 nm) were fabricated using thermal reduction of the mono-layer graphene oxide sheets in the presence of H_2_SO_4_ and HNO_3_. They demonstrated that the CDs-labeled Hela cells did not exhibit any reduction in PL during continuous excitation, indicating that CDs could be a potential substitute of dyes [101]. Zhu et al. used citric-acid, and ethylenediamine derived CDs for imaging MC3T3 cells [39]. A large scale synthesis of CDs from sucrose was reported by Chen et al. [102]. The as-prepared CDs were applied for 16HBE cells imaging at 488 nm [102]. After that Hu et al. synthesized blue luminescent N-doped CDs by hydrothermal treatment method for HeLa cells imaging. The CDs did not show any significant toxicity to the cells and were mostly localized in the cytoplasmic region [103]. Likewise, CDs synthesized from several other sources, like graphene oxide and DMF, Polycyclic aromatic hydrocarbon as well as graphite powder were also reported for in vitro cell imaging [104–106]. Folic acid based N-doped CDs were used by Wang et al. for imaging U87 glioma cells [107]. Polythiophene phenyl propionic acid derived red-emissive CDs were synthesized by Ge et al. [108], and used for both in vitro and in vivo imaging. For in vitro bioimaging, HeLa cells were treated with the CDs which showed red fluorescence localized in the cytoplasm when excited at 543 nm. They also intravenously injected CDs in the HeLa-tumor bearing mice and observed that the CDs were mostly accumulated inside the tumor due to enhanced permeation and retention (EPR) effect. Based upon their results, they concluded that the as-synthesized CDs could be applied for both fluorescent imaging as well as photoacoustic imaging agent [108]. In the same year, Krishna et al. used citric acid, PEG diamine, and glycerin to synthesize CDs and functionalize them with digitonin for cholesterol detection and imaging [109]. A mixture of phosphoric acid, citric acid, and ethylenediamine was used by Parvin et al. to synthesized N, P co-doped CDs via hydrothermal treatment and used for bioimaging of RAW 264.7 cells [110]. Borse et al. used urea, and polyethylene glycol (PEG) derived CDs for bioimaging in L929 cells. The CDs were localized in the cell cytoplasm and showed excitation-dependent multicolor emission [111]. Yang et al. fabricated F-doped CDs from the mixture of citric acid, sodium fluoride and urea, and used for imaging in both in vivo and in vitro models. They observed that CDs showed red fluorescence in the cytoplasm of glioma C6 cells when excited at 530 nm. In case of in vivo imaging, they injected CDs in the nude mice bearing xenograft tumor and checked the whole body fluorescence at an excitation wavelength of 530 nm and an emission wavelength of 600 nm. It was observed that the CDs were mostly accumulated within the tumor region due to the EPR effect [112].

**Table 5:** Role of CDs in bioimaging application

S. No.	Source molecule	Color	Application (bio-imaging)	Refs. No.
1.	Carbon soot	Blue–yellow	HepG2 cell	[40]
2.	Activated carbon	Blue/yellow/green	COS-7 cells	[94]
3.	Graphene oxide and DMF	Green	MG-63 cell	[93]
4.	Citric acid, AEAPMS and silica	Blue	BGC823 cell	[82]
5.	Glucose, monopotassium phosphate	Green	HepG2 cell	[52]
6.	Carbon fibers	Green	T47D Cell	[95]
7.	Carbon nanotubes and graphite	Yellow	In vivo NIR fluorescence imaging in mice	[3]
8.	Graphite rods and hydrazine	Yellow	Neurospheres cells, pancreas progenitor cells, and cardiac progenitor cells were performed	[96]
9.	CX-72 carbon black	Green	MCF-7 cell	[97]
10.	Glycerol, Polyethylenimine (*PEI*)	Blue/green/red	COS-7 cell	[9]
11.	Glycine	Green	MCF-10A, MCF-7 cells	[98]
12.	Glucose and TTDDA	Green	HeLa, MCF-7, NIH-3T3 cells	[99]
13.	Glycerol solvent	Blue	HeLa cell	[100]
14.	Graphene oxide and ammonia	Green	HELA cell	[101]
15.	Citric acid and ethylenediamine	Blue	MC3T3 cell	[39]
16.	Sucrose and oil acid	Green	16HBE cell	[102]
17.	Graphene oxide and ammonia	Blue	HeLa cell	[103]
18.	Graphene oxide and Dimethylformamide	Green	HeLa cell	[104]
19.	Polycyclic aromatic hydrocarbon	Green	MCF-7 cell	[105]
20.	Graphite powder	Green/blue	A549 cell	[106]
21.	Folic acid	Blue, Green	U87 glioma cell	[107]
22.	Polythiophene phenyl propionic acid	Red	HeLa cell imaging and diagnosis	[108]
23.	Citric acid, PEG diamine, and Glycerin	Blue	Cholesterol imaging	[109]
24.	Citric acid, phosphoric acid, and ethylene diamine	Red, green	RAW 264.7 cells, PA and FL imaging of mice tumors	[110]
25.	Urea, polyethylene glycol (PEG)	Blue	L929 cells	[111]
26.	Citric acid, urea and sodium fluoride	Red	Glioma C6 cells	[112]

### Drug/gene delivery

**Fig. 3:**
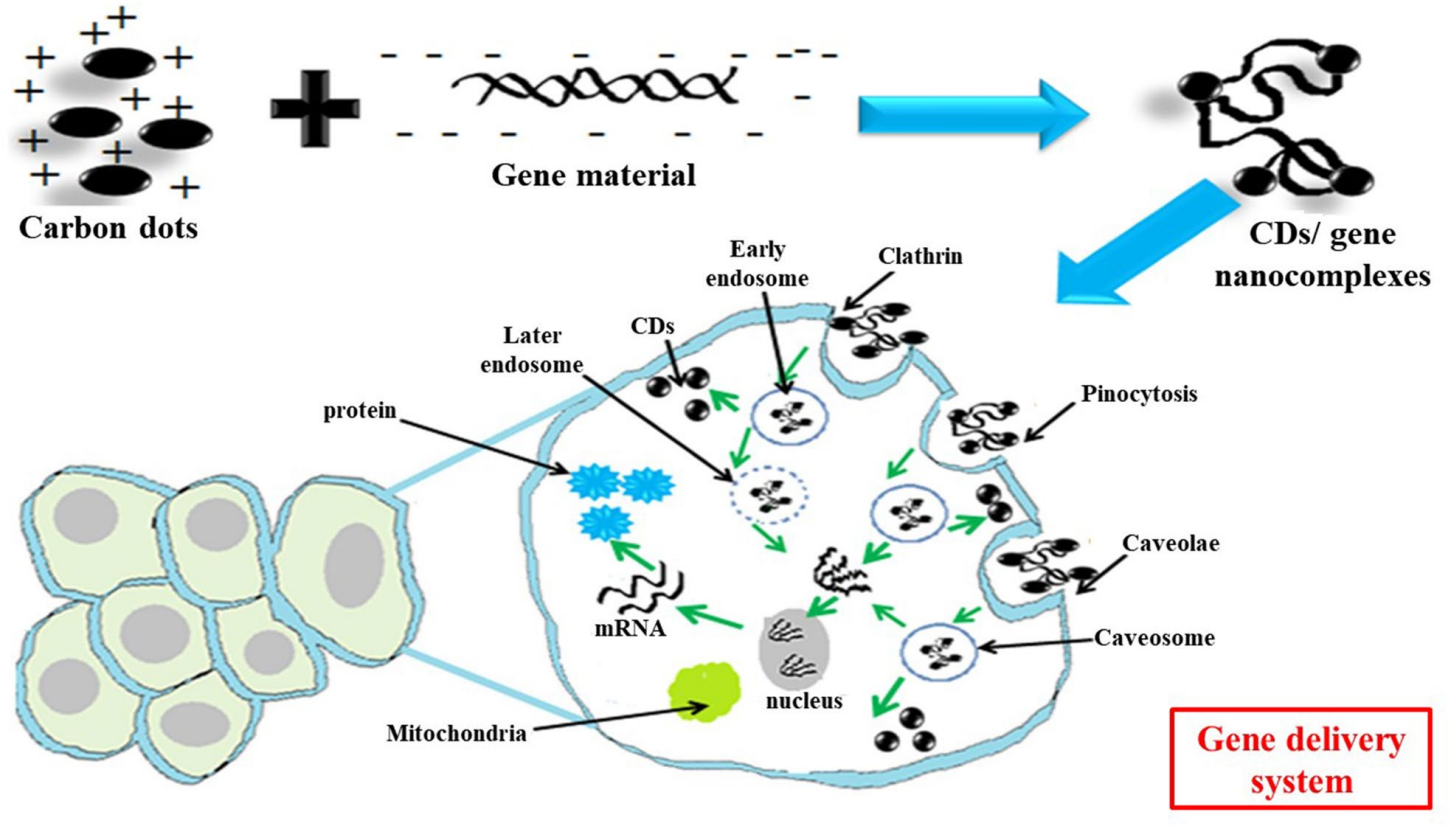
General mechanisms of gene delivery via CDs: here CDs bind with gene materials via electrostatic interactions, enter into the cells via endocytosis and release the payloads into nucleus

CDs can be used as a vehicle for drug/gene delivery due to their synthesis from cheap sources, facile surface functionalization, tiny size, and higher biocompatibility (Table 6). Besides, the drug/gene delivery pathway is also tracked due to the intrinsic fluorescence nature of CD; therefore considered as an excellent alternative to other fluorescent dyes or semiconductor nanoparticles (Figs. 2, 3) [113]. In order to deliver the anticancer drug, doxorubicin (DOX) into cancer cells, Zhou et al. used fluorescent CD-gated mesoporous silica nanoparticles (MSPs) as a pH-responsive drug carrier and bioimaging system. The as-prepared CDs@MSPs were biocompatible and showed strong fluoresce both in vitro and in vivo. DOX-loaded CDs@MSPs entered into cancer cells via endocytosis, showed a pH-responsive drug release behavior in mildly acidic condition and enhanced cytotoxicity in Hela cells [114]. After that Yang et al. fabricated β-cyclodextrin (βCD), oligoethylenimine (OEI) and Phosphoric acid based green luminescent CDs for a drug delivery system. They further functionalize CDs with HA (hyaluronic acid) for active targeting onto tumor cells. The nanoparticles were non-cytotoxic and exhibited green fluorescence in H1299 cells. They also demonstrated that DOX-loaded CD nanocomplexes exhibited higher cytotoxicity towards H1299 cells than that of free DOX [115]. Wang et al. used CDs/DOX nanocomplexes as the image-guided drug delivery system. They synthesized the CDs form citric acid and o-phenylenediamine, and the positively charged DOX was loaded on the surface of negatively charged CDs through electrostatic interactions. However, the fluorescence of CDs decreased after loading with DOX, while after the release of DOX from the surface of CDs, fluorescence was regained and thereby acted as an image-guided drug delivery system [116]. Further, the CDs/DOX nanocomplexes showed higher cytotoxicity towards cancerous cells (HeLa) compared with normal cells (L929, mouse fibroblast cells).

**Table 6:** Role of CDs in drug/gene delivery system

S. No.	Source molecule	Ligand attached	Drug/gene delivery	Cell type	Refs. No.
1.	EDTA	Mesoporous silica nanoparticles (MSPs)	DOX	HeLa	[114]
2.	Sorbitol and sodium hydroxide	Folic acid	DOX	HeLa	[118]
3.	β-Cyclodextrin (βCD), oligoethylenimine (OEI) and Phosphoric acid	OEI/CD	DOX	H1299	[115]
4.	citric acid and *o*-phenylenediamine	–	DOX	HeLa, mouse fibroblast cells (L929)	[116]
5.	Carbon nanopowder	Transferrin	DOX	Glioblastoma cells; CHLA-266, DAOY, CHLA-200 and SJGBM2 cells	[119]
6.	Urea and citric acid	carboxyl groups on CDs	DOX	HepG2 and HL-7702	[27]
7.	ATP (Adenosine Triphosphate) moreover, polyethyleneimine (PEI)	Hyaluronic acid (HA)	DOX	HeLa cells	[120]
8.	d-Glucose (2.5 mmol) and l-glutamic acid	Polydopamine coated	DOX	HeLa	[121]
9.	Citric acid and polyene polyamine (PEPA)		Oxaliplatin	Hepatic cancer cells	[122]
10.	Citric acid and ethylenediamine	–	DOX	L929, MCF-7 and CCK-8	[117]
11.	Citric acid and diethylenetriamine	PEG-(PAH/DMM)	Cisplatin	A2780	[11]
12.	Glycerol and polyethylenimine (PEI)	fc-rPEI (folate conjugated reducible PEI rPEI)	siRNA	H460	[123]
13.	Citric acid and tryptophan	PEI	siRNA	MGC-803	[60]
14.	Branched polyethyleneimine, Hyaluronic acid	Hyaluronate (HA) and polyethyleneimine (PEI) functionalized	DNA/RNA	Hela cells	[124]
15.	Polyethyleneimine and fluorinated diglycidyl ethers	Fluorine doped	siRNA/DNA	7702, A549 and HepG2	[125]
16.	Arginine and glucose	–	pSOX-9	Chondrogenic differentiation of mouse embryonic Fibroblasts	[76]

Recently Kong et al. fabricated a citric acid and ethylenediamine based CDs for drug delivery systems. According to their results they observed that DOX could be quickly loaded on CDs through electrostatic interaction and the CDs/DOX complexes showed better cellular uptake and antitumor efficiency on the breast cancer MCF-7 cells compared with free DOX [117]. Similarly, some other researchers have also used CDs loaded with DOX for simultaneous cell imaging and drug delivery systems [27, 114, 118–120]. Zheng et al. fabricated fluorescent CDs with an anticancer drug, oxaliplatin (CD-Oxa) via condensation reaction between the carboxyl groups of oxaliplatin derivative and the amino groups on the CD surface to increase its anticancer efficacy. They demonstrated that the as-synthesized CD-Oxa could enter into hepatic cancer cells and release the active drug under reducing environment. They also showed that the CDs were biocompatible towards normal fibroblast cells, whereas the CD-Oxa was cytotoxic towards hepatic cancer cells. They used the CDs and CD-Oxa as image guided drug delivery system for both in vitro and in vivo applications. They took hepatic cancer cells zenograft tumor mice model and found that intra-tumoral injection of the CD-Oxa effectively killed tumor cells and decreased the tumor volume with less systemic toxicity [122]. Later, Feng et al. used cisplatin (IV) pro-drug-loaded charge convertible CDs [CDs-Pt(IV)@PEG-(PAH/DMMA)] for image-guided drug delivery based upon the citric acid and diethylenetriamine as the precursor’s molecules.

The charge convertible CDs were obtained via further functionalizing the CDs with PEG-poly(allylamine) and polydimethylmaleic acid (PEG-(PAH/DMMA). From the in vitro experiments, they demonstrated that the charge convertible nano-carriers offer higher therapeutic capability in the tumor extracellular environment compared with normal physiological conditions. Besides, they also used zenograft tumor bearing mice model and found that intravenous injection of the nanoformulations exhibited better tumor cells inhibition capability with less systemic toxicity [11].

Wang et al. fabricated CDs based upon citric acid and tryptophan (as passivation agent) for imaging-guided survivin siRNA delivery in the gastric cancer cells. The as-prepared CDs were further coated with PEI which binds the negatively charged siRNA. The western blot and qRT-PCR experiments assured the survivin gene silencing via nanoparticle-mediated siRNA delivery into the MGC-803 cells. They also showed the secondary effects of survivin gene silencing in the MGC-803 cells, such as cell cycle arrest at the G1 phase and enhanced apoptosis [60].

Wu et al. used folate-conjugated reduction-sensitive polyethyleneimine passivated CDs for targeted EGFR and cyclinB1 siRNA delivery and targeting in H460 lung cancer cells. The combined siRNAs were released in reducing intracellular conditions and increased the anti-cancer activity in H460 cells [123]. Recently Cao et al. fabricated dual functional cationic CDs from glucose and arginine for imaging and SOX9 plasmid delivery in mouse embryonic fibroblasts (MEFs). They observed that CDs/pSOX9 nano-particles possessed low cytotoxicity towards MEFs, helped in the intracellular tracking, and SOX9-dependent chondrogenic differentiation [76]. Several other researchers have also worked with CDs, as non-viral gene delivery vectors [124, 125].

### Bio-sensing

**Fig. 4:**
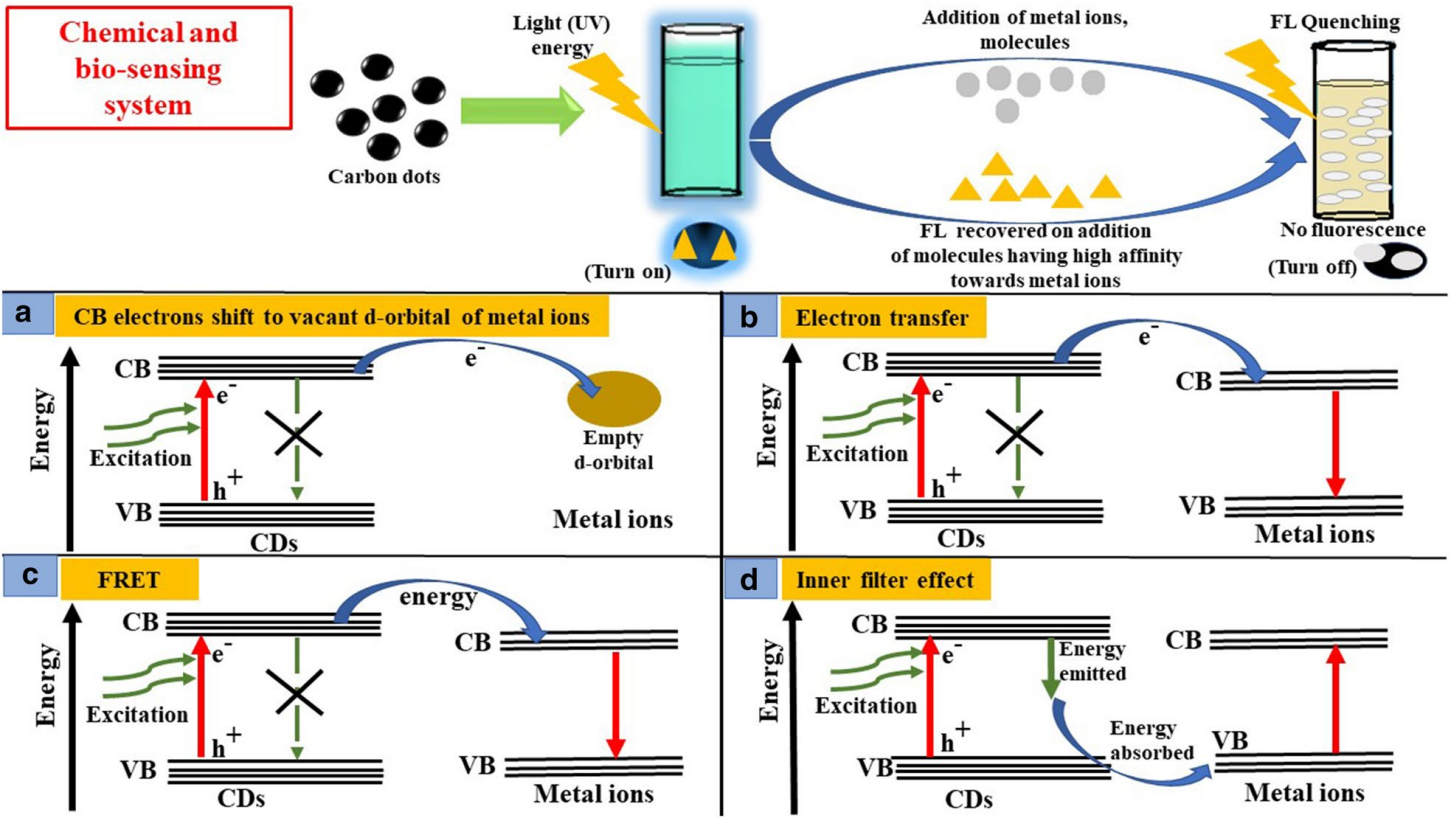
Role of CDs in chemical and bio-sensing via fluorescence quenching: the change in fluorescence intensity of CDs take place via different mechanisms, such as **a** Conduction band electrons of CDs shifts to low-lying vacant d-orbital of metal ions, **b** Electron transfer from CB of CDs to CB of metal ions, **c** Fluorescence resonance energy transfer (FRET), **d** Inner filter effect

CDs have also been employed by researchers as a bio-sensing and chemical-sensing nano-materials due to their unique properties like excitation-dependent emission, higher photostability, low cytotoxicity and better aqueous solubility (Table 7) [45]. The changes in their fluorescence property take place via different mechanisms, such as resonance energy transfer, inner filter effect, photo-induced electron and charge transfer [126]. These nanomaterials can be used for sensing of several biological molecules and intracellular metal ions, such of H_2_O_2_, Fe^3+^, C_6_H_12_O_6_, Vitamin B_12_, l-cysteine, galactose, etc. via changing their fluorescence intensity (Fig. 4). Wu et al. used l-glutamic acid derived CDs for effective detection of H_2_O_2_. The as-prepared CDs possessed peroxidase-like activity which was utilized for H_2_O_2_ detection in the presence of 2, 2^1^-azino-bis(3-ethylbenzothiazoline-6-sulphonic acid (ABTS) with the detection limit of 20 μM [17].

**Table 7:** Role of CDs in bio-sensing application

S. No.	Precursor molecule	Color	Application (bio-sensing)	Refs. No.
1.	l-Glutamic acid	Blue, green and red	H_2_O_2_	[17]
2.	SiCl_4_, hydroquinone	Blue	Fe^3+^, H_2_O_2_, and melamine	[29]
3.	BBr_3_, hydroquinone	Blue	H_2_O_2_ and glucose	[15]
4.	Dopamine and (3-aminopropyl) triethoxysilane, glycerol	Blue	Ag^+^	[127]
5.	Oxalic acid (OA) and urea	Blue	Fe^3+^ and Ag^+^	[128]
6.	Fullerenes (C60)	Blue	Fe^3+^	[130]
7.	Lactose and NaOH	Blue	Folic acid	[129]
8.	Galactose and *m*-aminophenyl boronic acid	Blue	Galactose	[131]
9.	Citric acid, aminoguanidine	Blue	Nitric oxide (NO)	[132]
10.	Citric acid and melamine	Blue	Glutathione	[134]

Qian et al. used hydroquinone, and SiCl_4_ derived CDs as the sensor for Fe^3+^, H_2_O_2_, and melamine. The detection of H_2_O_2_ was attained by an electron transfer mechanism between Si-doped CDs and H_2_O_2_, while both electron and energy transfer mechanisms were operating for detecting Fe^3+^. Formation of a stable adduct between H_2_O_2_ and melamine following melamine addition recovered the fluorescence and thereby acted as a sensitive detector for melamine as well [29]. Similarly, Shan et al. used B-doped CDs for selective detection of H_2_O_2_, and the detection limit was 10.0 mM. The same CDs could also be used for detection of glucose in the presence of glucose oxidase which produces H_2_O_2_ via oxidation of glucose [15]. Jiang et al. synthesized Si-CDs@DA (dopamine) via microwave-assisted method for the intracellular Ag^+^ detection via fluorescence quenching with the linear sensing range of 5 to 50 nM and detection limit of 2.5 nM [127]. In the same year, Lu et al. used water-soluble CDs obtained from oxalic acid (OA) and urea for effective detection of Fe^3+^ and Ag^+^ in biosystem. The CDs were capable of sensing Fe^3+^ with a linear range of 1.0 to 130 μM and Ag^+^ with a linear range of 0.50 to 200 μM [128]. Chen et al. fabricated fluorescent CDs from lactose and NaOH by simple heating. They observed that folic acid could quench the fluorescence of the as-prepared CDs upon binding and employed those CDs as the selective probe for folic acid detection in the human urine [129]. The detection of Fe^3+^ using fullerene (C60) derived CDs was demonstrated by Lan et al. [130]. They passivated the CD surface with hydroxyl and carboxyl groups which interact with Fe^3+^, thereby quenching the fluorescence [130]. Yang et al. introduced boronic acid functionalized CDs as the biosensor nanomaterials for detecting galactose. The boronic acid moiety present on the CD surface reacted with *cis*-diol units of galactose to give rise cyclic boronate esters, leading to quenching and selective detection of galactose in human urine [131]. Bhattacharya et al. also used aminoguanidine based CDs for nitric oxide (NO) detection via formation of azo dye [132]. Recently Iqbal et al. fabricated N-doped CDs from citric acid and melamine (passivation agent) and applied them for sensing glutathione and Hg^2+^ in the biological system via fluorescence quenching [133].

### Chemical-sensing

The interaction of metal ions and surface functional groups of CDs lead to the formation of new electron–hole recombination, with the help of energy transfer route and results in the change of the fluorescence nature of CDs (Fig. 4). This property of CDs is utilized for sensing different types of metal ions [7], such as Hg^2+^ [134], Ag^+^ [146], Cu^2+^ [12], Fe^3+^ [142] etc. (Table 8).

**Table 8:** Role of CDs in chemicals sensing application

S. No.	Precursor molecule	Application (chemical-sensing)	Refs. No.
1.	Ethylenediaminetetra acetic acid (EDTA)	Hg2+	[134]
2.	Sodium citrate and citric acid	Hg^2+^	[13]
3.	Ammonium citrate and ethylenediamine	Hg^2+^	[54]
4.	Citric acid, NH_3_·H_2_O	Hg^2+^	[6]
5.	Sodium citrate and citric acid	Hg^2+^	[135]
6.	Phenolphthalein and ethylenediamine	Hg^2+^, lemon yellow dye, Fe^2+^ and H_2_O_2_	[136]
7.	Citric acid and triethylamine	Hg^2+^	[137]
8.	*o*-Phenylenediamine	pH, Hg^2+^, Cl^−^ and Cr^4+^	[138]
9.	Graphite rods	Fe^3+^	[139]
10.	Polycyclic aromatic hydrocarbon (PAH)	Fe^3+^	[105]
11.	Ethylene glycol	Fe^3+^	[143]
12.	Citric acid	Fe^3+^	[144]
13.	Citric, thiourea	Fe^3+^	[145]
14.	Cetylpyridinium bromide (CPB)	Fe^2+^	[140]
15.	Folic acid and 3-aminopropyl trimethoxy silane	Fe^3+^	[141]
16.	Phenylenediamine	Fe^3+^	[142]
17.	Citric acid	Fe^3+^ and I^−^	[155]
18.	d-Sorbitol	Fe^3+^	[156]
19.	CCl_4_ as a carbon and diamines as nitrogen precursors	Ag+	[146]
20.	Uric acid	Ag^+^ and Hg^2+^	[147]
21.	1,2-diaminobenzene	Ag^+^	[148]
22.	Citric acid and amino acid	Ag^+^	[149]
23.	uric acids	Ag^+^	[150]
24.	Citric acid and guanidine thiocyanate	Ag^+^	[151]
25.	Urea, polyethylene glycol (PEG)	Ag+	[111]
26.	Citric acid, poly(ethylenimine) for BPEI-CQDs	Cu^2+^	[12]
27.	Ammonium citrate and ethylenediamine	I^−^	[54]
28.	Sodium alginate	Ascorbic acid	[91]
29.	Ascorbic acid and glycol	Al^3+^ and F^−^	[152]
30.	Citric acid	Selenite (SeO_3_^2−^)	[153]
31.	Citric acid, and 1,6-diaminohexane dihydrochloride	Cr^6+^	[154]

The heavy metal ion, Hg^2+^ is highly toxic, and CDs are employed for Hg^2+^ detection on several occasions. Zhou et al. demonstrated the use of unmodified CDs for the detection of Hg^2+^ and biothiols (glutathione, cysteine, and homocysteine) with higher selectivity and sensitivity. They observed that the addition of Hg^2+^ to CDs caused fluorescence quenching. However, subsequent addition of biothiols to the Hg^2+^/CDs recovered the fluorescence via the removal of Hg^2+^ ions which has a high affinity towards thiol (–SH) groups [134]. After that Guo et al. synthesized CDs from sodium citrate/citric acid via hydrothermal treatment for the selective and sensitive detection of Hg^2+^ ions [13].

Similarly Li et al. and Zhang et al. fabricated N doped CDs and applied them for effective detection of mercury ions [6, 54]. Later, Xu et al. demonstrated the use of blue fluorescent Mn-doped CDs for the highly selective and sensitive detection of Hg^2+^ ions via fluorescence quenching with a nM detection limit due to its higher chemical affinity towards Hg^2+^ ions compared with various other metal ions. The fluorescence quenching by Hg^2+^ might be due to the electron transfer or energy transfer from CDs [135]. Pan at el. fabricated green fluorescent CDs from phenolphthalein and ethylenediamine for multi-mode sensing. They observed that the fluorescence emission of CDs was selectively quenched by Hg^2+^ ions (with detection limit 5.8 μM) due to the photo induced electron transfer mechanism between them. Furthermore, CDs exhibited significant fluorescent response to lemon yellow dye, H_2_O_2_ and Fe^2+^ with detection limits of 0.5, 1.1 and 1.2 μM respectively [136]. Wang et al. synthesized N-doped CDs from citric acid and triethylamine via hydrothermal treatment for rapid and selective detection of Hg^2+^ ions. They observed that the N-doped CDs could act as highly sensitive sensor to Hg^2+^ ions in tap water samples, with a detection limit of 2.8 nM [137]. Recently Li et al. fabricated blue/yellow emissive CDs from o-phenylenediamine as the multi-mode sensor materials and applied for the selective detection of Hg^2+^, Cl^−^ and Cr^4+^ ions. They observed that blue emission of CDs get quenched by Hg^2+^ (turn off) and recovered on addition of Cl^−^ ions (turn on). Furthermore, blue/yellow fluorescence of CDs get quenched significantly on addition of Cr^4+^ ions [138]. Zhang et al. synthesized graphitic CDs as fluorescent material for selective and sensitive detection of the Fe^3+^ ions. They observed that the hydroxyl and carboxyl groups present on the surfaces of CDs help in interaction with Fe^3+^ ions and thereby enabling CDs to act as the fluorescence sensor for detecting Fe^3+^ ions with a detection limit of 2 nM [139]. Recently the detection of Fe^2+^ with the help of CDs based upon cetyl pyridinium bromide as the source molecule was reported by Kaur et al. [140]. They observed that the fluorescence intensity of CDs was decreased gradually with the addition of Fe^2+^ ions with increasing concentration. This technique is also used in determining the Fe^2+^ ions in syrup and iron tablets [140]. Wu et al. synthesized folic acid/3-aminopropyl trimethoxy silane based N, Si-doped CDs via one-step hydrothermal method and applied for sensing Fe^3+^ ions. They observed that Fe^3+^ ions could be detected using the as-synthesized CDs with a detection limit of 3.8 nM [141]. Sun et al. fabricated red-emitting CDs via microwave irradiation of phenylenediamine and applied for detection of Fe^3+^ ions in aqueous solutions with a detection limit of 15 nM. They demonstrated that fluorescence of CDs get quenched significantly in presence of Fe^3+^ due to electron transfer mechanism [142]. Similar articles relating to the detection of Fe^3+^ were also published in the past few years [105, 143–145, 155, 156]. Qian, et al. synthesized blue fluorescent N-doped CDs from CCl_4_ and diamines for the detection of Ag^+^ ion. Their results suggested that fluorescence intensity of the as-prepared CDs was inversely proportional to the pH values ranging from 5.0 and 13.5, indicating the possibility of using those CDs as a pH indicator. Besides, they also used the CDs for detection of Ag^+^, which increased the fluorescence intensity of CDs upon binding with the N-atoms on their surface [146]. Binding of Ag^+^ ions with the amino groups on the CDs surface leads to positive charges on CDs which decreased the collision among them and increased the fluorescence. Borse et al. also used N-doped CDs for the ‘turn-off’ dynamic fluorescence-based sensing of Ag^+^ ions with detection within the concentration range of 1–1000 μM [111]. Ren et al. (2017) synthesized blue fluorescent N-doped CDs from uric acid for the selective detection of Ag^+^ and Hg^2+^ ions with detection limit of 1 and 4.8 nM respectively. Besides, the quenching of fluorescence of N-doped CDs could be recovered on the addition of EDTA [147]. Li et al. fabricated 1,2‐diaminobenzene based N-doped CDs as a sensitive detection probe for Ag^+^ with a detection limit of 0.5 nM [148]. Wang et al. employed citric acid and amino acid derived carbon dots (CDs)-gold nanoparticles (AuNPs) hybrid for sensing Ag^+^ ions in presence of glutathione. They demonstrated that on the addition of Ag^+^ ions to a solution containing CDs-AuNPs and GSH, solution color changes from red to blue due to the aggregation of AuNP. This method could be used to detect Ag^+^ ions with a detection limit of 50 nM [149]. Recently, Qin et al. synthesized blue fluorescent CDs via pyrolysis of uric acids and N, S co-doped CDs from citric acid and guanidine thiocyanate via hydrothermal method for the selective detection of Ag^+^ ions [150, 151]. Lin et al. used citric acid and poly(ethylenimine) based CDs in a zeolitic imidazolate framework and act as a selective detecting agent for Cu^2+^ ions with a detection limit of 80 pM. The intense blue emission of CD-nanocomposites was quenched following the addition of Cu^2+^ ions [12]. Zhang et al. fabricated citric acid based N doped CDs (NCDs) as the chemical sensing materials and applied for “turn off” detection of Hg^2+^ ions. That NCDs-Hg^2+^ system was further used for the selective and sensitive detection of l-cysteine via “turn on” mechanism with a detection limit of 79 nM [6]. Fong et al. synthesized blue fluorescent CDs from sodium alginate for the detection of Fe^3+^ and ascorbic acid. They showed that Fe^3+^ ions decreased the fluorescence intensity (turn off) of the CDs, which was recovered by ascorbic acid addition (turn on) [91]. Sun et al. employed ascorbic acid, and glycol derived green fluorescent CDs for sensitive and selective detection of Al^3+^ ions with the detection limit of 0.39 µM via fluorescence enhancement response. They demonstrated that the combination of Al^3+^ ions with the hydroxyl groups on CDs at nearly neutral pH resulted in the creation of much more surface state of the CDs, thereby increasing the fluorescence intensity. However, they also used the formed CDs-Al^3+^ ion system for selective and sensitive detection of F^−^ anions with a detection limit of 0.14 µM via fluorescence “on–off” mechanism due to the strong affinity of F^−^ ions towards Al^3+^ ions [152].

Devi et al. used nitrogen-rich ligands functionalized CDs for selective detection of selenite (SeO_3_^2−^) via fluorescence quenching mechanism (with the detection limit of 0.1 ppb) due to the formation of strong Se–N bonds [153]. N and S co-doped CDs were prepared by Chen et al. form citric acid and 1,6-diaminohexane dihydrochloride and employed those for Cr^6+^ detection with the detection limit of 0.86 μM. They proposed that the fluorescence quenching of CDs by Cr^6+^ ions were due to the inner filter effect [154].

### Photocatalytic application

CDs exhibit potential applications in the field of photo-catalytic reactions due to their unique properties like better water solubility, very less toxicity, and high chemical stability compared with another common photocatalysts, such as ZnO, CdS, TiO_2_, etc. (Table 9). In addition, CDs can exhibit up-converted photoluminescence (UCPL) and photoinduced electron transfer properties which attract much interest in designing photocatalysts utilizing CDs [44]. Qu et al. fabricated S, N co-doped CDs/TiO_2_ composites for the degradation of Rhodamine B (RhB) as a visible light photocatalyst. They proposed that S, N: GQDs absorbed the visible light, and electronic excitation occurred from valence band (VB) to conduction band (CB). The electrons were then injected from the CB of CDs to CB of TiO_2_ (CDs acts as a sensitizer) and promoted the charge separation process. This charge separation process further generates superoxide and hydroxyl radicals which subsequently degraded the dye [157]. Yu et al. prepared CDs modified TiO_2_ (CDs/P25) nanocomposites for efficient photocatalytic hydrogen evolution under both UV and visible light irradiation. When CDs/P25 was exposed to the UV light, CDs acted as electrons reservoir and improved the efficient separation between photo-induced electron–hole pairs. However, under visible light irradiation, CDs acted as a sensitizer and promote electron excitation in P25 [158]. Wang et al. used vitamin-C derived CDs/TiO2 nanocomposites for hydrogen production from water. They demonstrated that the efficient electron transfer ability and up-conversion properties of CDs contributed to the enhanced photocatalytic behavior of TiO2 [159]. Martins et al. synthesized N-doped CDs/TiO2 composite with improved photocatalytic activity [160]. Cai et al. prepared S, N co-doped CDs/g-C_3_N_4_ nanocomposites for the high catalytic performance in degrading RhB under visible light irradiation. They observed that S, N doped GDs/g-C_3_N_4_ nanocomposites exhibit better photocatalytic activity than pure g-C_3_N_4_ due to electron transfer from the CB of g-C_3_N_4_ to CB of S, N doped GDs/g-C_3_N_4_ (CDs act as an electron reservoir), results in the efficient separation of photogenerated electrons and holes [161]. Recently Zhang et al. fabricated CDs/La_2_Ti_2_O_7_ nanocomposites for the degradation of RhB. The as-synthesized CDs absorbed visible light and converted it into UV light (due to its up-conversion properties) which was utilized by La_2_Ti_2_O_7_ for charge separation. Besides, the CDs were also capable of accepting electrons from La_2_Ti_2_O_7_, thereby promoting efficient separation of photogenerated electrons and holes [162]. Similar kind of photodegradation of RhB was also reported by several other researchers using CDs nanocomposites [163–166]. The zinc oxide reduced graphene oxide (ZnO-RGO) nanocomposites were synthesized by Li et al. for the RhB photodegradation and CO_2_ photoreduction. They observed that ZnO-RGO nanocomposites showed enhanced photocatalytic activity and the main reasons were: (1) RGO acted as electron acceptor, i.e. photoexcited electrons of ZnO transferred from its CB to the CB of RGO; (2) RGO absorbed a large number of dye molecules on their surface due to pi-stacking interaction [167]. They found that under the visible light irradiation g-C_3_N_4_ could be utilized for the photoreduction of CO_2_ to organic fuels. The urea derived g-C_3_N_4_ showed better CO_2_ photoreduction due to their porous like structure and larger surface area than that of melamine derived g-C_3_N_4_ [168]. Sahu et al. fabricated Au-doped CDs from small acetic acid for the photoreduction of CO_2_ [169]. Cao et al. used Au or Pt-coated PEG-functionalized CDs as an active photocatalyst for the reduction of CO_2_. Surface functionalization with PEG increases the solubility of the catalysts allowing homogeneous reaction in the aqueous phase. The coated Au or Pt over the CD surface soaks up the photogenerated electrons, thereby disrupting the electron/hole recombination process [170].

**Table 9:** Role of in CDs in photocatalytic application

S. No.	Nanomaterial	Source molecules	Photocatalysis application/role of support	Refs. No.
1.	S, N doped GDs/TiO_2_	Citric acid for c-dots and urea/thiourea for N, S	Degradation of Rhodamine B	[157]
2.	CDs/Ag/Ag_2_O	Glucose	Rhodamine b	[174]
3.	CDs/g-C_3_N_4_	Citric acid, ethylenediamine	Degradation of Rhodamine B and tetracycline hydrochloride (TC-HCl)	[175]
4.	S, N doped GDs/g-C_3_N_4_	Citric acid and thiourea	Rhodamine B (RhB) degradation	[161]
5.	CDs/Bi_2_O_3_	l-Ascorbic acid	degradation of Rhodamine b	[165]
6.	Ultrafine amorphous iron oxyhydroxide/ultrathin g-C_3_N_4_	Urea	Degradation of Rhodamine B, methylene blue, and methyl orange	[166]
7.	CDs/La_2_Ti_2_O_7_	Vitamin C and ethanol	Rhodamine B (RhB)	[173]
8.	Reduced graphene oxide/ZnO	Graphite oxide	CO_2_ photoreduction	[178]
9.	g-C_3_N_4_	Urea or melamine	Conversion of CO_2_ into methanol	[168]
10.	Au-doped CDs	Carbon-based	CO_2_ Photoreduction	[169]
11.	PEG1500N-functionalized CDs with Au/Pt doping	Carbon-based	H_2_ generation, and CO_2_ photoreduction	[170]
12.	CDs/TiO_2_	Graphite	H_2_ generation	[170]
13.	CDs/TiO_2_	Vitamin C	H_2_ generation	[159]
14.	CDs	Citric acid	H_2_ generation	[85]
15.	N doped GDs-ZnNb_2_O_6_/g-C_3_N_4_ heterostructures	Urea for g-C_3_N_4_ and C_6_H_5_O_7_(NH_4_)_3_, NaOH for NGDs	H_2_ generation	[171]
16.	CDs/nitrogen-doped ZnO	Carbon black pigment	Methylene blue	[172]
17.	N doped CDs/TiO_2_	Glycerol and TTDDA	Degradation of methylene blue	[160]
18.	CDs/Ag/Ag_2_O	Glucose	Degradation of methylene blue	[174]
19.	Ultrafine amorphous iron oxyhydroxide/ultrathin g-C_3_N_4_ nanosheets	Urea	Methylene blue, and methyl orange	[166]
20.	Fe(III)/CDs	Oxidative coupling of Xylene by anhydrous FeCl3	H_2_O_2_ reduction	[162]
21.	CDs/nitrogen-doped ZnO	Carbon black pigment	Degradation of malachite green	[172]
22.	La/Cu/Zr/CDs	d-Fructose, NaOH	Degradation of ampicillin antibiotic, malachite green	[174]
23.	N doped CDs	Glucose and ammonia	Photodegradation of methyl orange	[175]
24.	Ultrafine amorphous iron oxyhydroxide/ultrathin g-C3N4 nanosheets	Urea	Methyl orange	[166]
25.	CDs/Bi_2_WO_6_	Citric acid, ethylenediamine	Degradation of methyl orange and bisphenol A	[176]
26.	CDs/ZnFe_2_O_4_	L-Ascorbic acid,, glycol, and deionized water	NO removal	[178]
27.	Pb–CDs–TiO_2_	Ascorbic acid and kollicoat	Degradation of RBX, CRB, and CNB dye	[177]
28.	Ag-CDs/g-C_3_N_4_	Citric acid, ethylenediamine	Naproxcen	[179]
29.	CDs/g-C_3_N_4_/MoO_3_	Citric acid, urea and dicyandiamide	Degradation of tetracycline (TC)	[180]

Martindale et al. fabricated citric acid derived CDs via straightforward thermolysis route and combined it with molecular Ni catalyst [Ni-bis(diphosphine)] for solar hydrogen production [85]. Yan et al. used N doped CDs-ZnNb_2_O_6_/g-C_3_N_4_ nanocomposite for photocatalytic hydrogen production. Under visible light irradiation, NGQDs absorbed light with wavelength ≥ 550 nm and converted them to shorter wavelength < 460 nm that was further used to excite g-C_3_N_4_ and to promote the generation of more electron–hole pairs. The electrons of g-C_3_N_4_ in CB were further injected into the CB of the ZnNb_2_O_6_ and trapped by the Pt nanoparticles [171]. Eco-friendly CDs/N-doped ZnO nanocomposites were used by Muthulingam et al. for the degradation of methylene blue, fluorescein and malachite green dyes under the sunlight irradiation. It was noticed that CDs helped in electron/hole separation in ZnO via accepting the electrons from its CB and also exerted anti-photocorrosion property due to its coating over the ZnO surface [172]. Chen et al. synthesized CDs/Ag/Ag_2_O nanocomposites as an excellent photocatalyst for pollutant degradation via incorporating the photoinduced electron transfer and up-converted properties of CDs [163]. Yang et al. prepared ultrafine iron oxyhydroxide/g-C3N4 nanocomposites heterojunctions as an efficient photocatalyst in the presence of visible light. They observed that the ultrafine nanocomposites helped to increase the visible light absorbance, shortened the band gap of photogenerated carriers, and also helped in the formation of a large number of heterojunctions with g-C_3_N_4_ [166]. Bourlinos et al. synthesized Fe(III)-functionalized CDs for catalytic decomposition of H_2_O_2_ and hydrogenation of olefin. It was observed that Fe(III)-functionalized CDs were more useful for the hydrogenation of electron withdrawing or donating olefin substrates than heterogeneous or homogeneous Fe(III)-based photocatalysts [173].

Mao et al. synthesized two types of g-C_3_N_4_ from melamine and urea via pyrolysis method.

**Fig. 5:**
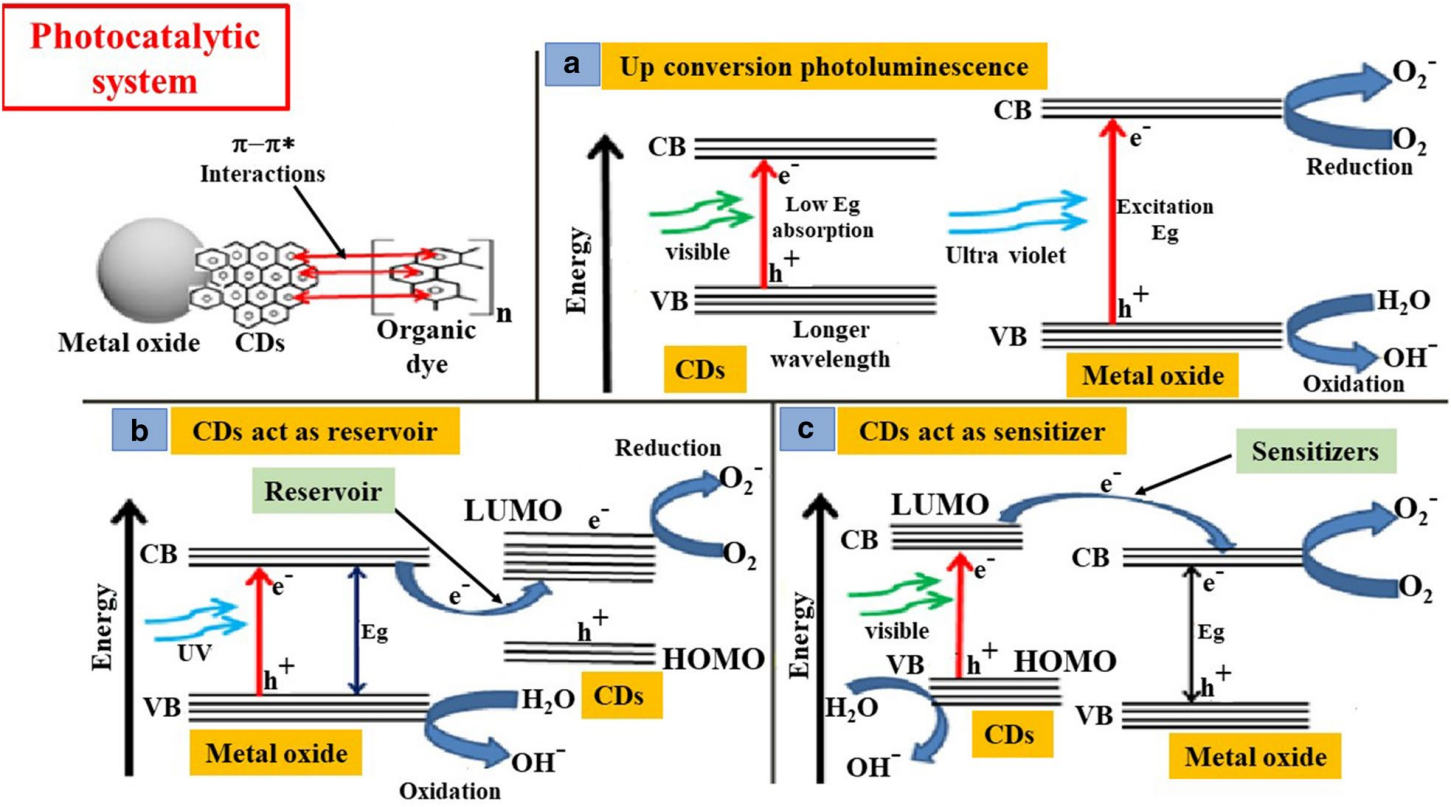
Role of CDs in photocatalytic applications: CDs help in photocatalysis via different mechanisms, such as **a** up conversion photoluminescence of CDs, **b** action of CDs as electron reservoir, **c** action of CDs as sensitizer. Furthermore, the π–π stacking between CDs and organic dye also enhances its degradation upon light irradiation

Several other researchers also used CDs nanocomposites for the photocatalytic degradation of ampicillin antibiotic [174], organic/industrial dyes [175–177], NO [178], naproxcen [179], and tetracycline [180]. The overall mechanisms of action behind the photocatalytic applications CDs have been depicted in Fig. 5.

### Photo-dynamic therapy (PDT) and Photo-thermal (PTT) therapy

Photo-dynamic therapy (PDT) and Photo-thermal therapy (PTT) are applied for the treatment of cancer using laser light (most often by near IR radiations). PTT refers to the use of NIR which is absorbed by a photoabsorber to generate local heat, and destroy diseased tissue. PTT offers several advantages over the conventional chemotherapy, radiotherapy, and surgery; therefore attracting much interests of the researchers in the field of cancer treatment. However, the effectiveness of nanomaterials as photothermal agent (gold nanostructures, graphene and graphene oxide) in PTT is still under consideration due to the difficulties in their synthesis, and high production cost. Unlike the PTT, PDT requires O_2_ to produce ROS and destroy targeted cells. In PDT, a photosensitizer is irradiated with laser light that produces ROS which ultimately destroys the cancer cells/tissue. Upon excitation with a suitable wavelength, photosensitizer gets excited from its ground singlet state to higher energy singlet state and suffers intersystem crossing to form long lived triplet state. The triplet state reacts with oxygen molecule, resulting in ROS that can effectively destroy cancer cells [181]. Recently CDs are intensively used by researchers in PTT and PDT due to its smooth and low-cost synthesis, facile surface functionalization and superior caring capacity, excellent biocompatibility and ability to convert absorbed light into heat due to a large number of pi electrons (Table 10). Both PDT and PTT are involving the use of light having less energy and hence is less injurious to healthy cells/tissue [113]. Ge et al. fabricated red-fluorescent CDs from polythiophene phenylpropionic acid for photoacoustic/FL (NIR) imaging and in vitro as well as in vivo photo-thermal therapy. They demonstrated that the as-synthesized CDs showed extensive cytotoxicity towards HeLa cells upon NIR laser irradiation. The CDs were also found to show in vivo PTT efficacy in HeLa tumor bearing mice without any sign of systemic toxicity [182]. Zheng et al. fabricated CDs from hydrophobic cyanine dye and poly(ethylene glycol) for NIR imaging and in vitro as well as in vivo photothermal therapy. The as-prepared CDs exhibit superior photothermal effect in HepG2 and CT26 cells upon NIR laser irradiation. The CDs were also effective in inhibiting tumor growth in CT26 xenograft Babl-c mice upon NIR laser irradiation [183]. Li et al. synthesized supra-CDs from citric acid, and urea via electrostatic interactions and hydrogen bonding. The as-prepared supra-CDs showed strong visible-NIR absorption with high photothermal conversion efficiency [184]. Beack et al. synthesized CDs-Ce6-HA, hyaluronic acid modified CDs conjugated with chlorine-Ce6 (Ce6, a photosensitizer) for targeted therapy of melanoma skin cancer. They observed that CDs conjugation to Ce6 increased the photodynamic reaction of Ce6, and produce more singlet oxygen than free Ce6. Transdermal administration of CDs-Ce6-HA resulted in suppression of B16F10 melanoma cells in tumor mice upon laser irradiation [185]. Yang et al. fabricated Mg/N doped CDs and used them as a carrier of Ce6. This CDs/Ce6 composite showed high fluorescence resonance energy transfer (FRET) efficiency and subsequently enhanced the PDT effect. They also demonstrated that CDs conjugation to Ce6 increased the production of singlet oxygen almost two times than the free Ce6, and a significantly enhanced PDT effect was observed in HepG2 cancer cells compared with free Ce6 [10]. Zheng et al. synthesized carbon nitride (C_3_N_4_) doped CDs and functionalized it with a targeting agent and a photosensitizer to increase the targeted PDT efficiency in solid tumor under hypoxic condition. They demonstrated that the nanocomposite produces oxygen inside the hypoxic tumor region via splitting of water upon light exposure, thereby producing ROS and showed enhanced PDT efficiency under both in vitro and in vivo conditions [186].

**Table 10:** Role of CDs in photo-dynamic therapy (PDT) and photo-thermal (PTT) therapy

S. No.	Source molecule	Ligand attached	Targeted cell type	Refs. No.
1.	Polythiophene phenylpropionic acid	–	Hela cells	[182]
2.	Diaminohexane and carboxylic group of Ce6	Ce6-HA (hyaluronate)	B16F10 melanoma	[185]
3.	Acrylic acid, 1, 2-ethylenediamine (EDA) and Mg(OH)_2_	Mg/N	HepG2	[10]
4.	Hydrophobic cyanine dye and poly(ethylene glycol)	–	HepG2, CT26	[183]
5.	Citric acid and urea	–	HeLa	[184]
6.	Dopamine	–	Hela cells	[181]
7.	Urea	Carbon nitride (C_3_N_4_)	4T1	[186]
8.	Citric acid and 5,10,15,20-tetrakis(4-aminophenyl)porphyrin	Cetuximab (C225)	HCC827 and MDA-MB-231 cells	[187]
9.	*m*-Phenylenediamine and l-cysteine	Protoporphyrin IX (PpIX)	HeLa	[188]
10.	EDTA·2Na and CuCl_2_	–	Murine melanoma (B16) cells	[189]

Wu et al. synthesized porphyrin implanted CDs from citric acid and 5,10,15,20-tetrakis(4-aminophenyl)porphyrin via selective pyrolysis treatment. They demonstrated that the as-prepared CDs could act as an active agent for photoacoustic molecular imaging under NIR irradiation. Furthermore, cetuximab-conjugated CDs exhibit superior photodynamic effect in HCC827 and MDA-MB-231 cancer cells as well as MDA-MB-231 tumor bearing mice [187].

**Fig. 6:**
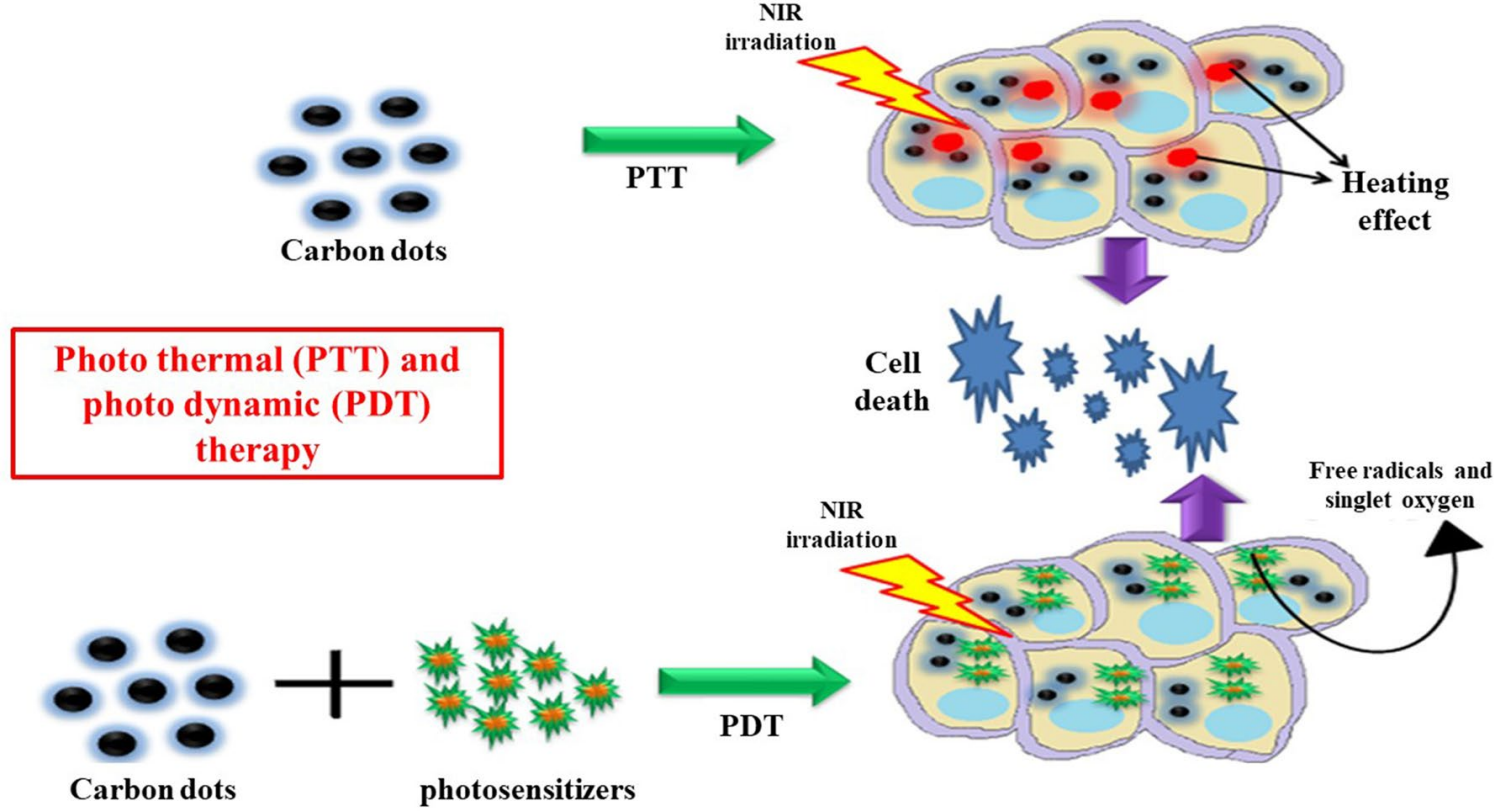
Role of CDs in Photo thermal (PTT) and Photo dynamic (PDT) therapy. PTT: after the cellular internalization of CDs, laser light irradiation (mostly near infrared) is used which is absorbed by the CDs to generate local heat and destroy diseased tissue. PDT: CDs carry a photosensitizer into the cellular system. Upon laser light irradiation, free radicals/reactive oxygen species are generated, leading to cancer cell death

Recently Hua et al. synthesized CDs from l-cysteine and m-phenylenediamine. The as-prepared CDs showed nucleus targeting property, thereby could be used as a nucleus staining agent. They further conjugated the CDs with protoporphyrin IX (a photosensitizer), which showed enhanced PDT efficacy in vivo without affecting healthy cells [188]. Guo et al. synthesized Cu, N co-doped CDs via one-step hydrothermal treatment using EDTA·2Na and CuCl_2_. They demonstrated that the as-synthesized Cu, N co-doped CDs significantly inhibited cancer (B16 melanoma) cell growth via photodynamic and photo-thermal therapy. In addition it could also act as an active agent in FL cell-imaging and IR thermal imaging to visualize in vivo and in vitro treatment process [189]. The Role of CDs in photo thermal (PTT) and photo dynamic (PDT) therapy has been depicted in Fig. 6.

## Conclusion and future perspective

The first report of CDs synthesis was registered in 2004 as the new fellow of carbon family and attained tremendous attention in the area of chemistry and biology due to their economical and facile synthesis methods, easy surface modification, excellent photoluminescence, and superior water solubility.

CDs can be extensively applied in photocatalytic reactions, in vitro, and in vivo bioimaging, drug-gene delivery system, chemical and biological sensing as well as in photodynamic and photothermal therapies. This review summarized the progress of research on CDs regarding its synthesis from small organic molecules and applications in biological as well as chemical field till date. Although significant development has been done in those areas over the past decade, still some difficulties need to be addressed related to the synthesis and applications of CDs as follows:

- The synthesis of CDs with uniform size distribution is somehow tricky. The size distribution plays a crucial role to decide its toxicity and fluorescence properties which may obstruct further biological applications, particularly in vivo. CDs reproducibility is also a big issue for clinical applications. Besides, different synthesis methods of CDs result in different QY, size, fluorescence color, etc. which obstruct CDs commercialization.
- Despite enormous research on CDs synthesis and its modifications, the real mechanism of CDs formation is not understood fully till date. Furthermore, simple and controllable surface modifications are still crucial problems, which may help for designing CDs with excellent photoluminescence property and other applications with high efficiency.
- Although CDs based chemical and, bio-sensing technology is well applicable in real life samples, but studies on several other toxic metal ions, like Cd^2+^, Mn^2+^, As^3+^, Po^3+^, etc. are still missing. Therefore, further research with those metal ions should be done to understand the full potential of CDs as a sensing agent.
- More efforts are needed to apply the CDs in the field of in vivo imaging, drug and gene delivery systems as well as cancer therapies to broaden the area of its applications. Furthermore, dual drug-gene delivery systems are not fully explored to date.
- Finally, the use of CDs in the area of energy storage is needed to be explored. Therefore, researchers should concentrate on those above-said issues and CDs will gain significant interest in the future undoubtedly after proper addressing such problems.

## Data Availability

Not applicable.
